# Bradley’s Lip and Cheek Holder

**Published:** 1866-06

**Authors:** 


### BRADLEY'S LIP AND CHEEK HOLDER.

This is a very ingenious appliance for holding up, and away from the teeth, the lip and cheek while the teeth are being filled It consists of a broad silver hook, to embrace the angle of the mouth or lip, attached to a neat morocco strap about an inch in width, and two feet long. The hook takes hold upon the lip, the strap passes over the head, and is held in the hand of the patient, by this means the lip is kept entirely away from the teeth, an additional piece, called the duct compressor, can be attached to the hook, and with a small pad of paper effectually closes the orifice of the duct of steno. The appliance is susceptable of a variety of applications which will be readily apparent very soon by use. All should have it.

